# Event‐Driven Type Design for Clinical Trials With Recurrent Events

**DOI:** 10.1002/sim.70742

**Published:** 2026-09-14

**Authors:** Jingwen Zhang, Satoshi Hattori

**Affiliations:** ^1^ Department of Biomedical Statistics, Graduate School of Medicine The University of Osaka Osaka Japan; ^2^ Integrated Frontier Research for Medical Science Division Institute for Open and Transdisciplinary Research Initiatives (OTRI), The University of Osaka Osaka Japan

**Keywords:** Andersen–Gill model, blinded continuous monitoring, frailty model, proportional rate model, sandwich variance

## Abstract

It is common practice in randomized clinical trials with the standard survival outcome to follow patients until a prespecified number of events have been observed, a type of trial known as the event‐driven trial. The event‐driven design ensures that the target power for a specified Type I error rate is achieved to detect the target hazard ratio, regardless of the specification of other quantities. To understand the treatment effect for chronic conditions, the analysis of recurrent events has gained popularity in randomized controlled trials, particularly large‐scale confirmatory trials. In the absence of within‐subject correlation among multiple events, a similar event‐driven design can be employed for recurrent event outcomes. On the other hand, in the presence of within‐subject correlation, one needs to model the correlation among recurrent events in evaluating power and setting the sample size. However, information useful in modeling the within‐subject correlation is limited at the design stage. Failing to properly account for correlation may lead to underpowered studies. We propose an event‐driven type design for recurrent event outcomes. Our method ensures the target power for the target treatment effect, regardless of the specification of other quantities, by monitoring the robust variance under the marginal rates/means model in a blinded manner. We investigate the operating characteristics of the proposed monitoring procedure in simulation studies. The results of simulation studies showed that the proposed blinded monitoring procedure controlled the power well so that the test possessed the target power and did not lead to serious inflation of the Type I error rate. Furthermore, we illustrate the proposed method using a real clinical trial dataset.

## 1 Introduction

In randomized clinical trials with the standard univariate time‐to‐event endpoint, the logrank test with the hazard ratio (HR) as the treatment contrast is widely and routinely used. The widespread acceptance of the logrank‐HR strategy is mainly due to the success of the clever and stable partial likelihood inference for the Cox proportional hazards model. Under the logrank‐HR strategy, the event‐driven design can be employed. Based on Schoenfeld's power formula of the logrank test [1, 2], one can derive the target number of events for a given treatment effect at the design stage. A notable feature of the event‐driven design is that the target power for the target treatment effect is ensured, regardless of the specification of nuisance quantities, provided the analysis is conducted once the prespecified number of events is observed. Furthermore, since the time of the statistical analysis can be determined without knowledge of the allocation of treatment to subjects, an event‐driven study can be implemented with blinded treatment codes. Thus, the event‐driven design should contribute to the broad applicability of the logrank‐HR strategy and minimize the risk of operational biases.

The advent of advanced therapies and innovative drugs has reshaped the clinical landscape. Many diseases that used to be fatal have now evolved into manageable chronic conditions characterized by multiple nonfatal events. This paradigm shift has motivated many researchers to think beyond the univariate time‐to‐event endpoint, choosing recurrent events as the primary endpoint for randomized controlled trials [3, 4, 5]. Recurrent events are defined as events that can occur repeatedly in the same patient over time. Compared with standard survival analysis, recurrent event analysis offers the potential to capture disease burden more effectively, increase statistical power, and reduce sample size [6, 7, 8].

Andersen and Gill [9] generalized the Cox proportional hazards model to recurrent events. The Andersen–Gill (AG) model is intensity‐based, modeling the instantaneous incidence of a new event at a specific time *t* given the individual's entire event history up to *t*. The AG model assumes that the time increments between events for a patient are independent, corresponding to a Poisson process. Here, we refer to the assumption as the Poisson‐type assumption in the rest of this paper. With the Poisson‐type assumption, Bernardo and Harrington [10] derived the expected number of events under a homogeneous Poisson process. Using their formula, an event‐driven design can be conducted as in the standard survival analysis. The Poisson‐type assumption imposes a specific dependence structure of recurrent events. However, the within‐subject dependence of event times can be more complicated. The risk of ignoring such within‐subject dependence includes the inflation of Type I error [11]. Thus, in the analysis and design of clinical trials with recurrent events, the within‐subject dependence among events should be handled appropriately. Lin et al. [12] developed the robust sandwich variance estimator, which is valid in the presence of within‐subject dependence.

On the other hand, however, almost all existing sample size calculation methods that account for within‐subject correlation provide formulas for the number of subjects rather than events under some strong assumptions about the probabilistic structure of the recurrent events. If the focus of trials is only the event counts over a prespecified period, sample size formulas proposed by Tango [13] and Li et al. [14] can be considered. Both discussed the violation of the Poisson‐type assumption because data from recurrent events often exhibit overdispersion relative to a Poisson process. Tango recommended using a robust sandwich variance estimator from a conditional Poisson model, while Li et al. assumed that the variance is proportional to the mean of the event counts. Modeling events via a homogeneous Poisson process, Cook [15] proposed parametric calculations for the expected number of subjects or trial duration under the Poisson regression model. Additionally, he modified his formula by introducing subject‐specific random effects for modeling event counts. Matsui [16] developed asymptotic sample size formulae for a nonparametric test comparing the rate functions of two groups under a mixed Poisson process (MPP) with a Weibull baseline intensity, explicitly incorporating frailty to account for within‐subject dependence. Tang and Fitzpatrick [17] provided power and sample size formulas for robust Wald tests in the AG model assuming a negative binomial distribution for event counts, covering superiority, noninferiority, and equivalence settings.

All the methods discussed in the last paragraph set the target number of subjects. As an exceptional event‐driven study for recurrent events, Ingel and Jahn‐Eimermacher [18] extended Bernardo and Harrington's method [10] to overdispersed data by adopting a frailty‐based inflation factor (IF) derived from the decomposition of the robust variance under the AG model proposed by Al‐Khalidi et al. [19]. However, the target number of events derived by Ingel and Jahn‐Eimermacher [18] depended on specific assumptions for recurrent event processes at the design stage, which are vulnerable to misspecification of the level of dispersion, baseline rate functions, follow‐up schemes, and so forth. Even if the target treatment effect is fixed, it may not ensure the target power because prior knowledge of the probabilistic structure of recurrent events is limited.

To address this particular issue, several strategies for sample size re‐estimation have been proposed to mitigate parameter misspecification at the design stage. Cook [20] proposed a two‐stage design to revise the sample size with complete or partial blinding, in which an EM algorithm was used to re‐estimate the constant baseline intensity function and the frailty variance. Likewise, Ingel and Jahn‐Eimermacher [18] proposed a blinded sample size recalculation method to update the specification of the frailty variance. However, their methods still rely on the MPP. Besides, the reestimation of the frailty variance only uses data from an internal pilot study, which is a small subset of the recurrent events within a limited time window. To make full use of accumulating data and get rid of assumptions regarding event rate forms and within‐subject dependence, we propose a blinded continuous monitoring procedure built on the semiparametric marginal mean/rate model. The monitoring threshold is computed using only the anticipated treatment effect, which mirrors the simplicity of classic event‐driven designs. The robust variance proposed by Lin et al. [12] under the semiparametric model is used to drive the stopping criteria and is estimated nonparametrically from pooled data.

The remainder of this article is organized as follows. We first present the notation and statistical models for analyzing recurrent events, and then provide the power formula and details of the blinded monitoring procedure in Sections 1, 2, 3. In Section 4, we assess the proposed method's operating characteristics and compare it with a conventional event‐driven design and a re‐estimation procedure in simulation studies. Section 5 illustrates the practical implementation using a dataset from a chronic disease trial. Finally, we conclude with a brief discussion.

## 2 Preliminary

### 2.1 Setting and Notation

We consider a two‐arm clinical trial in which the primary outcome is a recurrent event. A total of n patients are randomized to an experimental or a control group. The treatment allocation for subject i is defined by a binary random variable Z with P(Z=1)=π, taking the value 1 for the experimental group and 0 for the control group. Each subject can experience the event of interest multiple times. Denote $N^{*}(t)$ the underlying counting process for events of interest, which is defined as the number of events observed over the interval [0,t]. Events are subject to right‐censoring by the censoring time C, and $N(t)=N^{*}(t\wedge C)$ is the observed number of events. Y(t)=I(C≥t) denotes the at‐risk process. Hence, the observed data consist of n independently and identically distributed copies of {N(·),Y(·),Z}. They are denoted by $\left\{N_{i}(\cdot ),Y_{i}(\cdot ),Z_{i}\right\}$ for i=1,…,n, where the subscript of i represents the quantity for the ith subject. We consider a staggered entry study; we suppose that subjects enter the study within an accrual period of duration $\tau_{a}$ years from the date of study initiation. Then, the follow‐up duration is supposed to be subject‐specific.

### 2.2 AG Model and Marginal Rate Model

We begin with the AG model [9], though our primary focus is the marginal rate model. It is an extension of the Cox proportional hazards model to recurrent event data. Although, the AG model allows us to incorporate multiple explanatory variables, we focus on a comparison of two groups, including a single covariate Z. The intensity function $\lambda_{Z}(t)$ is defined by

$$E\{dN^{*}(t)|{ℱ}_{t}\}=\lambda_{Z}(t)dt,$$

where ${ℱ}_{t}$ is a filtration generated by $\left\{N^{*}(s),Z:0\leq s\leq t\right\}$. The AG intensity model is defined by

(1) $$\lambda_{Z}(t)=\lambda_{0}(t)\exp(\beta Z),$$

where $\lambda_{0}(t)$ is an unspecified continuous baseline intensity function, and β is an unknown regression coefficient representing a treatment effect, with true value denoted by $\beta_{0}$. This formulation implies that $N^{*}(t)$ is a time‐transformed Poisson process and possesses the independent increment structure [12]. Let τ be a constant satisfying P(C≥τ)>0. One can estimate the regression coefficient by maximizing the partial likelihood function [21]. It is obtained by solving the score equation U(β)=0, where

(2) $$U(\beta )=\overset{n}{\underset{i=1}{\sum }}\int_{0}^{\tau }(Z_{i}-\bar{Z}(\beta ,u))dN_{i}(u),$$

$S^{\left(r\right)}(\beta ,t)=n^{-1}\sum_{i=1}^{n}Y_{i}(t)Z_{i}^{r}\exp(\beta Z_{i})$, 0≤t≤τ and $\bar{Z}(\beta ,t)=\frac{S^{\left(1\right)}(\beta ,t)}{S^{\left(0\right)}(\beta ,t)}$. The resulting estimator is denoted by $\hat{\beta }$. Andersen and Gill [9] showed that $n^{1/2}(\hat{\beta }-\beta_{0})$ is asymptotically zero mean normal with covariance $A^{-1}$,

(3) $$A=E\left[\int_{0}^{\tau }{\left(Z-\bar{z}(\beta_{0},u)\right)}^{2}Y(u)\exp(\beta_{0}Z)d\Lambda_{0}(t)\right],$$

where $\Lambda_{0}(t)=\int_{0}^{t}\lambda_{0}(u)du$ is the cumulative baseline intensity function and $\bar{z}(\beta ,t)$ the stochastic limit of $\bar{Z}(\beta ,t)$.

As mentioned, the standard AG model requires the independent increment structure, which may not be satisfied in practice. The marginal rate model allows us to make a similar inference without assuming the Poisson assumption and the independent increment structure by modeling the marginal rate of recurrent events [22, 23, 24]. It is defined by

(4) $$E\{dN^{*}(t)|Z\}=d\mu (t|Z)=\exp(\beta_{0}Z)d\mu_{0}(t),$$

where μ(t|Z) is the mean function of recurrent events given treatment allocation and $\mu_{0}(\cdot )$ is an unknown continuous baseline marginal mean function for recurrent events. The treatment effect is measured by $\beta_{0}$, the log‐rate ratio between two treatment groups.

Let the solution to U(β)=0 with (2) still be denoted by $\hat{\beta }$ for the marginal rate model. Lin et al. [12] showed that it remains consistent even for the marginal rate model. On the other hand, the asymptotic variance of the estimator should be modified. To account for the within‐subject dependence, a sandwich‐type robust variance should be used for $Var(\hat{\beta })$. Lin et al. further justified that $n^{1/2}(\hat{\beta }-\beta_{0})$ is asymptotically zero mean normal with covariance $A^{-1}\sum A^{-1}$, where

(5) $$A\equiv E\left[\int_{0}^{\tau }{\left(Z-\bar{z}(\beta_{0},u)\right)}^{2}Y(u)\exp(\beta_{0}Z)d\mu_{0}(t)\right],$$

(6) $$\sum =E\left[\int_{0}^{\tau }(Z_{1}-\bar{z}(\beta_{0},u))dM_{1}(u)\int_{0}^{\tau }(Z_{1}-\bar{z}(\beta_{0},v))dM_{1}(v)\right],$$

and $M_{i}(t)=N_{i}(t)-\int_{0}^{t}Y_{i}(u)\exp(\beta_{0}Z_{i})d\mu_{0}(u)$. Note that for the standard AG model (1) with the Poisson assumption, $\mu_{0}(t)=\Lambda_{0}(t)$ and A in (3) agree with that in (5). Therefore, under the AG model, A=∑ holds, and the covariance is reduced to $A^{-1}$ as argued. However, it does not hold in general for the marginal rate model. The variance of $n^{1/2}(\hat{\beta }-\beta_{0})$ is consistently estimated by ${Â}^{-1}\hat{\sum }{Â}^{-1}$, where

$$Â=\frac{1}{n}\overset{n}{\underset{i=1}{\sum }}\left[\int_{0}^{\tau }{\left\{Z_{i}-\bar{Z}(\hat{\beta },u)\right\}}^{2}Y_{i}(u)\exp(\hat{\beta }Z_{i})d{\hat{\mu }}_{0}(t)\right],$$

$$\hat{\sum }=\frac{1}{n}\overset{n}{\underset{i=1}{\sum }}\left[\int_{0}^{\tau }\{Z_{i}-\bar{Z}(\hat{\beta },u)\}d{\hat{M}}_{i}(u)\int_{0}^{\tau }\{Z_{i}-\bar{Z}(\hat{\beta },v)\}d{\hat{M}}_{i}(v)\right],$$

where ${\hat{M}}_{i}(t)=N_{i}(t)-\int_{0}^{t}Y_{i}(u)\exp(\hat{\beta }Z_{i})d{\hat{\mu }}_{0}(u)$ and

$${\hat{\mu }}_{0}(t)=\int_{0}^{t}\frac{d\sum_{i=1}^{n}N_{i}(u)}{nS^{\left(0\right)}(\hat{\beta },u)}.$$

Denote ${\hat{v}}^{2}=n^{-1}{Â}^{-1}\hat{\sum }{Â}^{-1}$. Then, the robust Wald‐type test can be made by the test statistic $\hat{\beta }/\hat{v}$ referring to the standard normal distribution under the null hypothesis $\beta_{0}=0$.

## 3 Event‐Driven Type Design for Recurrent Events

### 3.1 Event‐Driven Design for the Standard AG Model

For standard univariate survival analysis, in which only a single event is observed per subject, or for first‐event analysis in the context of recurrent events, the event‐driven design is widely used in practice. Instead of targeting the total number of subjects, the event‐driven design focuses on the target number of events. Based on the Schoenfeld formula [1, 2], the target number of events for the two treatment groups is set, and the statistical analysis is conducted when the target number of events is observed. In practice, the number of subjects should be specified. It is typically defined by specifying the accrual and follow‐up durations, the survival function of the control group, and the target HR. This procedure applies to the standard AG model for recurrent events with the Poisson assumption [10].

Suppose the AG model (1) holds. To obtain a simple power formula, an additional assumption is that the censoring time C is independent of Z [25]. Bernardo and Harrington [10] assumed that $\beta_{0}$ was small, and the asymptotic variance $A^{-1}$ of $\sqrt{n}(\hat{\beta }-\beta_{0})$ was approximated by ${\left\{\pi (1-\pi )L/n\right\}}^{-1}$ or the variance of $\hat{\beta }$ was approximated by ${\left\{\pi (1-\pi )L\right\}}^{-1}$. Then, with $\beta_{0}$ as the target treatment effect to detect, one can set the target number of events so that the Wald‐type test, or equivalently the logrank test, achieves the target power given the significance level. To be specific, suppose that a two‐sided test will be performed at significance level α with given statistical power 1−γ. Let $z_{1-\alpha /2}$ and $z_{1-\gamma }$ be the 1−α/2 and 1−γ percentiles of the standard normal distribution, respectively. The required number of events in the two treatment groups in total is given by

(7) $$L_{0}=\frac{1}{\pi (1-\pi )}{\left(\frac{z_{1-\frac{\alpha }{2}}+z_{1-\gamma }}{\beta_{0}}\right)}^{2}.$$

For a balanced design, in which π=1/2, the required number of events is calculated as $4\cdot {\left(z_{1-\alpha /2}+z_{1-\gamma }\right)}^{2}/\beta_{0}^{2}$.

Formula (7) extends the Schoenfeld formula for univariate survival analysis and enables us to conduct an event‐driven study; likewise, the statistical analysis is performed when the target number of events is observed. Note that the formula for the number of events does not rely on any quantities other than the treatment effect $\beta_{0}$, given the significance level and the target power. It implies that as long as the statistical analysis is conducted with the target number of events, the target power is ensured for the target treatment effect. Bernardo and Harrington [10] proposed a method for determining the number of subjects to enroll to achieve the target number of events within the planned duration of the study. Some strong assumptions on the probabilistic structure of the recurrent events are required for this calculation. However, it should be emphasized that misspecification of the probabilistic structure of recurrent events may prolong the study duration but does not render studies underpowered. It is a very important feature, since information about the probabilistic structure of recurrent events at the design stage is very limited.

### 3.2 Existing Sample Size Planning Methods With the Robust Variance

On one hand, as argued in Section 3.1, the Poisson‐type assumption enables event‐driven designs for recurrent events using the AG model. On the other hand, the Poisson‐type assumption might be too strong because recurrent events are usually correlated within each subject, and the occurrence of a new event tends to be associated with previous events. In this subsection, we review methods for deriving the required number of events or subjects without the Poisson assumption, using robust variance to account for within‐subject dependence in sample size planning.

Matsui [16] proposed the sample size calculation method for the logrank test for recurrent events. Although, the test is nonparametric, determining the sample size requires specifying several parameters. Matsui proposed specifying them based on the MPP model. Song et al. [26] proposed a sample size formula with the covariate‐adjusted robust logrank test for recurrent events. Their motivation was to provide a formula that did not rely on parametric models. Although, their formula consists solely of nonparametric quantities, they are difficult to estimate at the design stage unless individual participant data from previously conducted studies with a similar design are available. Thus, the resulting sample size should be subject to uncertainty at the design stage. Following Song's derivation, Tang and Fitzpatrick [17] also gave sample size formulas for the robust Wald test from the AG model for different study goals. To determine sample size, they provided analytic expressions for the robust variance under the assumption of a Weibull or piecewise constant event rate function.

All the methods introduced in the last paragraph proposed formulas for calculating the number of subjects. On the other hand, based on the robust score test, Ingel and Jahn‐Eimermacher [18] proposed a formula to calculate the target number of events in the two treatment groups. Assuming the follow‐up duration, denoted by $\bar{\tau }$, is common over subjects, Ingel and Jahn‐Eimermacher [18] proposed a formula for the target number of events as

(8) $$L=\frac{1}{\pi (1-\pi )}\left\{1+Var(\lambda )\frac{\bar{\tau }}{E(\lambda )}\right\}\cdot {\left(\frac{z_{1-\frac{\alpha }{2}}+z_{1-\gamma }}{\beta_{0}}\right)}^{2},$$

where λ is an individual‐specific random constant hazard over time, and the expectation and the variance are taken over the study population. The formula enables us to take an event‐driven design for recurrent events. Comparing the formulas (7) and (8), one observes that the required number of events for the robust test (8) equals the required number of events under the Poisson assumption $L_{0}$, defined by (7), multiplied by the IF,

$$\text{IF}=\left\{1+Var(\lambda )\frac{\bar{\tau }}{E(\lambda )}\right\}.$$

Again, this IF was derived assuming a constant individual event rate and equal follow‐up time for all subjects [19]. However, it is impossible to specify E(λ) and Var(λ) without specifying some probabilistic structure of the recurrent events. Taking the event process as a MPP, given an unobservable subject‐specific frailty $\eta_{i}$, the conditional intensity function of this subject may be modeled as

(9) $$\lambda_{i}\left(t|Z_{i},\eta_{i}\right)=\eta_{i}\cdot \lambda_{0}(t)\cdot \exp(\beta_{0}Z_{i}),$$

where $\lambda_{0}(t)$ is a common baseline intensity function of the target population. The term $\eta_{i}$ is often referred to as a frailty in survival analysis, indicating heterogeneity in the risk of event recurrence within the population. Typically, $\eta_{i}$ is assumed to follow a distribution with mean 1 and variance θ [27], where larger θ indicates greater heterogeneity. Under this frailty model, Ingel and Jahn‐Eimermacher [18] proposed to use the following formula, generalizing (8) to time‐varying baseline rate functions,

(10) $$\begin{array}{cc} & L=\frac{1}{\pi (1-\pi )}\left\{1+\theta \cdot \mu_{0}(\bar{\tau })\cdot \left(\frac{1+\exp\left(2\beta_{0}\right)}{1+\exp\left(\beta_{0}\right)}\right)\right\}\cdot {\left(\frac{z_{1-\frac{\alpha }{2}}+z_{1-\gamma }}{\beta_{0}}\right)}^{2}, \end{array}$$

where $\mu_{0}(t)$ is the baseline mean function for recurrent events and $\mu_{0}(\bar{\tau })=\int_{0}^{\bar{\tau }}d\mu_{0}(s)$. Then, the number of subjects is given by

(11) $$n=\frac{L}{\mu_{0}(\bar{\tau })\cdot (\pi \exp(\beta_{0})+(1-\pi ))}.$$

To determine the number of events L, one needs to specify the frailty variance θ and the baseline mean function $\mu_{0}(t)$. Misspecification of these quantities would lead to a poor estimate of power. Thus, even if the statistical analysis is conducted when the target number of events calculated by (10) is attained, the power may be less than the target.

Ingel and Jahn‐Eimermacher [18] presented the impact of a misspecified frailty variance θ on power. To address this issue, Ingel and Jahn‐Eimermacher [18] proposed taking an internal pilot study approach to re‐estimate θ and update the target number of events and subjects. At the design stage, given the target treatment effect $\beta_{0}$, the target number of events and sample size are set using (10) and (11) with an initial guess for $\mu_{0}(\bar{\tau })$ and $\theta_{1}$. At the internal pilot study conducted when the observed number of events reaches $L_{0}$, a semiparametric frailty model is fitted to the pooled data to update $\theta_{1}$, and then the number of events is updated to $L_{2}$ with the formula (10) and the sample size is also updated to $n_{2}$ with the formula (11). Note that Ingel and Jahn‐Eimermacher [18] used the same $\mu_{0}(\bar{\tau })$ as that at the design stage. The number of subjects is increased up to $n_{2}$, and the final analysis is conducted when the updated target number of events $L_{2}$ is attained.

### 3.3 The Proposed Procedure: Blinded Monitoring of $v^{2}$

Let us consider the power of the robust Wald test based on the mean rate model (4). Consider testing the null hypothesis $H_{0}:\beta_{0}=0$ against a local alternative $H_{1}:\beta_{0}=\frac{\delta }{\sqrt{n}}$, where δ is a constant. As argued in Section 2.2, $\sqrt{n}(\hat{\beta }-\beta_{0})$ approximately follows $N(0,A^{-1}\sum A^{-1})$ or the distribution of β is approximated by $N(\beta_{0},v^{2})$, where $v^{2}=n^{-1}A^{-1}\sum A^{-1}$. For a given two‐sided significance level α, the power is given by

(12) $$power=\Phi (-z_{1-\alpha /2}-\beta_{0}/v)+1-\Phi (z_{1-\alpha /2}-\beta_{0}/v),$$

where Φ(·) is the cumulative distribution function of the standard normal distribution. Thus, by setting the target power on the left side of Equation (12), the corresponding variance $v^{2}$ can be calculated for a prespecified treatment effect $\beta_{0}$ at the design stage. The resulting $v^{2}$ is denoted by $v_{\text{target}}^{2}$.

From Slutsky's theorem, the asymptotic distribution of the robust Wald test $\hat{\beta }/v$ does not change if A and ∑ in $v^{2}$ are replaced with their consistent estimators. We consider consistent estimators under the local alternative. As argued in Section 2.2, Â has the same stochastic limit as π(1−π)L/n. In the Appendix A, we show that the limit of $\hat{\sum }$ can also be written in a form free of treatment allocation $Z_{i}$ when π=1/2. Then, the variance $v^{2}$, under a 1:1 randomization ratio, can be estimated in a blinded manner by

(13) $${\hat{v}}_{\text{blind}}^{2}={\left\{\frac{L}{4}\right\}}^{-1}\cdot \left(\frac{1}{4}\overset{n}{\underset{i=1}{\sum }}{\left[N_{i}(\tau )-{\hat{\mu }}_{0}(\tau \wedge C_{i})\right]}^{2}\right)\cdot {\left\{\frac{L}{4}\right\}}^{-1}.$$

As the study proceeds, the maximum follow‐up duration τ increases, as does the number of events in the two treatment groups, L. Thus, by continuing the follow‐up, the target power can be achieved with a sample size chosen as n. We propose to determine the timing of the statistical analysis by monitoring ${\hat{v}}_{\text{blind}}^{2}$ with (13). That is, the statistical analysis is conducted when ${\hat{v}}_{\text{blind}}^{2}$ attains the $v_{\text{target}}^{2}$ for the target power. More specifically, the following procedure is proposed.

Step 1 Set a two‐sided significance level α, target power, and the log‐rate ratio $\beta_{0}$ as the minimum clinically relevant treatment effect.
Step 2 Set the number of subjects with some most likely assumptions about the probabilistic structure of the recurrent events. For example, one may set the target number of subjects n, assuming a MPP as done by Ingel and Jahn‐Eimermacher, outlined in Section 3.2.
Step 3 Calculate the target variance $v_{\text{target}}^{2}$ for the setting in Step 1 with the power formula (12).
Step 4 Monitor ${\hat{v}}_{\text{blind}}^{2}$ in a blinded manner continuously as the study proceeds.
Step 5 Once ${\hat{v}}_{\text{blind}}^{2}\leq v_{\text{target}}^{2}$, conduct the final analysis using the marginal rate model.

The proposed procedure suggests choosing the timing of the final analysis adaptively by monitoring ${\hat{v}}_{\text{blind}}^{2}$ or, equivalently, the predicted power based on (12) with $v={\hat{v}}_{\text{blind}}$ to attain the target power. In Step 2, one may use any existing method discussed in Section 3.2 to calculate the number of subjects. To note, when the number of accumulated events is small, or the follow‐up duration is short, the resulting ${\hat{v}}_{\text{blind}}^{2}$ tends to be unstable. We consider initiating the monitoring process either after a preset time has elapsed or after a predetermined number of events has been reached. To address the instability of ${\hat{v}}_{\text{blind}}^{2}$, particularly in the early phase of monitoring, we recommend attaching a bootstrap confidence interval to inform the timing of the final statistical analysis. See Section 5.

## 4 Simulation Studies

### 4.1 Overview

The simulation studies consist of two settings. In the first setting, the sample sizes are calculated when the planning assumptions of the parameters are fulfilled. In the second setting, we specify the sample sizes using misspecified parameters at the design stage. The focus of the second setting is to test the proposed method's ability to maintain statistical power when certain sample size planning assumptions are misspecified.

We evaluated the proposed blinded monitoring procedure in terms of the ability to achieve the target power and control the Type I error probability. Six months after a trial was initiated, the monitoring procedure was conducted daily. The timing of the statistical analysis was determined by monitoring ${\hat{v}}_{\text{blind}}^{2}$, rather than by L. As stated in Step 5 in Section 3.3, the statistical analysis was conducted once ${\hat{v}}_{\text{blind}}^{2}$ agreed with $v_{\text{target}}^{2}$ of the target power.

We compared the performance of the proposed method with two other methods based on Ingel's formulas (10) and (11). One was the fixed design, in which the target number of events and the corresponding subjects were determined based on planning assumptions. The other was the internal pilot study approach introduced in Section 3.2. Since, our focus is on event‐driven type designs, we considered a modified version of the proposal by Ingel and Jahn‐Eimermacher [18]. We performed an internal pilot study to obtain ${\hat{\theta }}_{2}$ and updated the target number of events calculated at the design stage by $L_{2}$, while keeping the initial sample size unchanged. The final analysis of the re‐estimation procedure was conducted when the observed number of events reached $L_{2}$. For all three methods, we used the same sample size n.

In Section 4.2, we provide details on the simulation settings and data generation for the simulation studies. In Section 4.3, we summarize the results when the specification at the design stage is correct. In Section 4.4, the results of misspecified cases are presented. In Section 4.5, we present an additional simulation study to examine the impact of non‐administrative censoring.

### 4.2 Data Generation and Settings

We simulated a randomized controlled trial to compare the treatment effect between an experimental and a control group. All subjects were assigned randomly to either group by $Z_{i}∼\mathrm{Bin}(1,0.5)$. We defined a baseline mean function as a Weibull‐type one, $\mu_{0}(t)=\lambda t^{\nu }$. We set λ=1. The parameter ν described the time trend of event rates: ν=1 corresponded to a constant rate; ν<1 implied that the event occurred less frequently over time, while ν>1 meant that the event rate increased with time. To account for the within‐subject event times correlation, we applied the model (9) with the frailty following a gamma distribution with both shape and rate parameters set to $\frac{1}{\theta }$ for the individual event rate. θ={0,0.5,1} represented homogeneous, moderately heterogeneous, and highly heterogeneous individual event times among subjects, respectively. Event times were generated using an inverse transformation method and a recursive algorithm proposed by Jahn‐Eimermacher et al. [28]. All subjects entered the trial uniformly between 0 and $\tau_{a}$ years and were followed for at least $\tau_{f}$ years. Different choices of $\left(\tau_{a},\tau_{f}\right)$ would result in varied mean follow‐up durations. To examine the impact of different accrual and follow‐up schemes on the performance of the proposed method, we considered three combinations of $\tau_{a}$ and $\tau_{f}$ while keeping the planned trial duration, $\tau_{a}+\tau_{f}$, fixed at 3 years (calendar time). The planned follow‐up duration was subject‐specific and follows a uniform distribution on $\left[\tau_{f},\tau_{a}+\tau_{f}\right]$. Therefore, when calculating the target number of events and sample size at the design stage, $\mu_{0}(\bar{\tau })$ in formulas (10) and (11) was replaced by the baseline mean function for recurrent events $\mu_{0}(t)$ and was averaged over the uniform distribution of follow‐up durations. Additionally, since we allowed for possible prolongation of follow‐up, we generated event times beyond the planned 3 years for each subject. We set the longest follow‐up duration to 15 years. We set the target power to 0.8 and the two‐sided Type I error rate to 0.05. For power evaluation, we took $\beta_{0}=\log(0.8)$ whereas for Type I error evaluation, we used $\beta_{0}=0$. Empirical power and Type I error rate were estimated from 2000 simulated datasets.

In the first setting of the simulation study, when the sample size planning assumptions were correctly specified at the design stage, each parameter combination $\left\{\lambda ,\nu ,\theta ,(\tau_{a},\tau_{f})\right\}$ was used to calculate the sample size n for all three designs and the target number of events L at the design stage. The same parameters would be used to generate datasets.

In the second setting of the simulation study, we considered three scenarios of planning assumption misspecifications that might not fit the model used to generate the datasets. To clarify, in each scenario, a true parameter was used to generate the data, while an assumed parameter was used to determine the sample size at the design stage. In scenario‐1, we evaluated the impact of ignoring frailty variance when planning sample sizes. We generated the datasets with the frailty variance $\theta_{\text{true}}=\{0,0.1,0.2,0.3\}$, but (n,L) was calculated using $\theta_{\text{assumed}}=0$ at the design stage. In scenario‐2, similar to the first case, the research team may have considered estimating heterogeneity but ended up misspecifying it. The datasets were generated with $\theta_{\text{true}}=0.5$ while (n,L) was determined using $\theta_{\text{assumed}}=\{0.3,0.4,0.5,0.6,0.7\}$. In scenario‐3, the impact of a misspecified baseline rate function was investigated. To this end, the data were generated from a MPP with a Weibull‐type baseline mean function for $\nu_{true}=\{0.5,1,2\}$. At the design stage, we determined (n,L) with $\nu_{\text{assumed}}=1$, rendering a constant event rate.

### 4.3 Validity of the Proposed Monitoring Procedure

Table 1 summarizes the empirical power, Type I error rate, and the distribution of the analysis time. For the fixed design, the analysis time was measured by the calendar years when L events were observed. Here, the calendar years implied years from the enrollment of the first subject into the study. For the re‐estimation procedure, we reported the distribution of the updated target number of events $L_{2}$ and the corresponding time when $L_{2}$ was reached. Likewise, for the proposed monitoring procedure, aside from the calendar years when $v_{\text{target}}^{2}$ was attained, we also reported the corresponding number of events at the final analysis. In general, when the sample size planning assumptions were met, the empirical power of the proposed method was close to 0.8 and comparable to that of the fixed design and the re‐estimation procedure. The Type I error probability using the proposed monitoring procedure remained close to the nominal level of 0.05. Compared to the re‐estimation procedure, the proposed method yielded a wider interquartile range (IQR) of the number of events at the final analysis in all scenarios. In most cases, the proposed method yielded a similar analysis time to the planned trial duration, around 3 years. Exceptions occurred when the baseline rate function increased over time (ν=2), and heterogeneity was present (θ>0). The fixed design and the re‐estimation procedure showed reduced power in these cases, with empirical power ranging from 0.711 to 0.799. The results might suggest potential violations of the underlying assumptions to derive the IF in (10), such as unequal follow‐up durations and significant variability in the individual expected number of events $\mu_{i}(t)=\eta_{i}\cdot \mu_{0}(t)$ [19]. As a result, the target number of events and sample size might be underestimated. In contrast, the proposed method maintained the power close to 0.8. However, it prolonged the analysis time compared to the two comparators: The median analysis time extended up to 3.96 years (Q1, Q3: 3.61, 4.51), with a corresponding median event count about twice that of the L derived at the design stage (fixed: 3123 vs. re‐estimation: 3123 vs. proposed: 6550).

**TABLE 1:** The operating characteristics of the proposed monitoring procedure compared with a fixed event‐driven design and a re‐estimation procedure of the target number of events when sample size planning assumptions are correctly specified at the design stage.

					Ingel and Jahn‐Eimermacher [18]							Proposed			
					Fixed			Re‐estimation				Monitoring			
							Analysis time: Median (Q1, Q3)			Analysis time: Median (Q1, Q3)				Analysis time: Median (Q1, Q3)	
ν	θ	$\left(\tau_{a},\tau_{f}\right)$	*n* ^a^	*L* ^a^	Power	Type I error	Calendar time (years)	Power	Type I error	Calendar time (years)	# of events	Power	Type I error	Calendar time (years)	# of events
0.5	0	(1, 2)	445	631	0.796	0.047	2.98 (2.85, 3.12)	0.801	0.047	3.05 (2.91, 3.23)	633 (632, 636)	0.795	0.050	3.04 (2.76, 3.33)	637 (609, 667)
		(1.5, 1.5)	470	631	0.795	0.049	3.00 (2.88, 3.12)	0.808	0.048	3.05 (2.92, 3.22)	633 (632, 635)	0.799	0.049	3.03 (2.81, 3.29)	636 (608, 666)
		(2, 1)	502	631	0.792	0.053	3.00 (2.89, 3.10)	0.797	0.049	3.04 (2.92, 3.17)	633 (632, 634)	0.791	0.048	3.02 (2.84, 3.23)	634 (610, 663)
	0.5	(1, 2)	764	1085	0.818	0.048	3.01 (2.87, 3.14)	0.808	0.048	3.05 (2.75, 3.39)	1094 (1029, 1163)	0.811	0.052	3.02 (2.66, 3.46)	1088 (1009, 1173)
		(1.5, 1.5)	790	1061	0.797	0.056	3.00 (2.89, 3.12)	0.806	0.062	3.04 (2.75, 3.33)	1070 (1007, 1135)	0.802	0.056	3.05 (2.74, 3.43)	1074 (1002, 1156)
		(2, 1)	822	1034	0.791	0.048	3.00 (2.90, 3.10)	0.796	0.045	3.02 (2.81, 3.28)	1044 (989, 1101)	0.791	0.048	3.05 (2.81, 3.34)	1046 (984, 1117)
	1	(1, 2)	1084	1539	0.801	0.043	3.00 (2.87, 3.14)	0.790	0.054	3.03 (2.71, 3.37)	1548 (1454, 1646)	0.802	0.051	2.97 (2.52, 3.62)	1530 (1388, 1712)
		(1.5, 1.5)	1109	1490	0.803	0.060	3.00 (2.88, 3.12)	0.801	0.044	3.04 (2.76, 3.37)	1497 (1417, 1596)	0.799	0.058	3.05 (2.68, 3.55)	1510 (1380, 1662)
		(2, 1)	1141	1436	0.808	0.062	3.00 (2.90, 3.11)	0.787	0.054	3.03 (2.81, 3.30)	1447 (1369, 1535)	0.805	0.060	3.10 (2.79, 3.51)	1476 (1354, 1606)
1	0	(1, 2)	281	631	0.801	0.042	3.00 (2.93, 3.06)	0.813	0.051	3.05 (2.96, 3.16)	634 (632, 661)	0.802	0.044	3.03 (2.87, 3.20)	640 (604, 679)
		(1.5, 1.5)	312	631	0.796	0.052	2.99 (2.93, 3.06)	0.804	0.050	3.04 (2.96, 3.13)	633 (632, 655)	0.797	0.052	3.02 (2.89, 3.17)	639 (604, 676)
		(2, 1)	351	631	0.782	0.048	2.99 (2.94, 3.05)	0.795	0.043	3.03 (2.96, 3.11)	633 (632, 652)	0.793	0.048	3.02 (2.90, 3.14)	638 (605, 674)
	0.5	(1, 2)	600	1350	0.808	0.054	3.00 (2.93, 3.07)	0.802	0.040	3.04 (2.85, 3.21)	1367 (1275, 1461)	0.814	0.055	3.05 (2.81, 3.35)	1374 (1247, 1538)
		(1.5, 1.5)	632	1278	0.788	0.048	3.00 (2.94, 3.06)	0.809	0.048	3.03 (2.87, 3.20)	1300 (1210, 1383)	0.795	0.051	3.11 (2.87, 3.34)	1336 (1205, 1466)
		(2, 1)	670	1206	0.788	0.048	3.00 (2.94, 3.05)	0.779	0.046	3.02 (2.89, 3.16)	1216 (1144, 1287)	0.799	0.052	3.15 (2.96, 3.35)	1296 (1184, 1419)
	1	(1, 2)	920	2069	0.797	0.052	3.00 (2.93, 3.06)	0.798	0.052	3.02 (2.84, 3.21)	2084 (1950, 2232)	0.806	0.052	3.09 (2.73, 3.50)	2130 (1855, 2488)
		(1.5, 1.5)	951	1925	0.796	0.049	3.00 (2.94, 3.07)	0.775	0.044	3.03 (2.86, 3.21)	1951 (1828, 2091)	0.810	0.051	3.14 (2.86, 3.50)	2042 (1805, 2357)
		(2, 1)	990	1781	0.783	0.044	2.99 (2.94, 3.05)	0.785	0.054	3.02 (2.88, 3.17)	1799 (1686, 1917)	0.792	0.048	3.24 (3.00, 3.56)	1991 (1786, 2292)
2	0	(1, 2)	111	631	0.780	0.056	3.00 (2.96, 3.04)	0.801	0.060	3.05 (2.99, 3.14)	646 (632, 704)	0.782	0.058	3.05 (2.91, 3.18)	656 (593, 726)
		(1.5, 1.5)	134	631	0.781	0.056	3.00 (2.95, 3.04)	0.801	0.056	3.04 (2.98, 3.12)	636 (632, 692)	0.792	0.060	3.04 (2.92, 3.16)	652 (594, 714)
		(2, 1)	162	631	0.788	0.050	3.00 (2.96, 3.04)	0.795	0.052	3.04 (2.99, 3.11)	635 (632, 676)	0.791	0.052	3.04 (2.93, 3.15)	651 (592, 713)
	0.5	(1, 2)	431	2452	0.781	0.050	3.00 (2.96, 3.03)	0.774	0.051	3.02 (2.88, 3.13)	2478 (2248, 2718)	0.795	0.051	3.17 (2.90, 3.55)	2786 (2250, 3626)
		(1.5, 1.5)	454	2141	0.758	0.058	3.00 (2.96, 3.03)	0.755	0.058	3.00 (2.90, 3.10)	2151 (1980, 2326)	0.793	0.059	3.32 (3.08, 3.65)	2771 (2286, 3474)
		(2, 1)	482	1877	0.749	0.048	3.00 (2.96, 3.04)	0.755	0.043	3.00 (2.90, 3.09)	1865 (1730, 2040)	0.812	0.049	3.53 (3.30, 3.87)	2922 (2419, 3689)
	1	(1, 2)	750	4273	0.799	0.052	3.00 (2.97, 3.04)	0.798	0.056	3.02 (2.91, 3.14)	4329 (3955, 4724)	0.810	0.052	3.28 (2.89, 3.87)	5282 (3890, 7776)
		(1.5, 1.5)	773	3650	0.757	0.050	3.00 (2.96, 3.04)	0.756	0.054	3.01 (2.91, 3.12)	3693 (3378, 4009)	0.797	0.048	3.65 (3.26, 4.25)	6007 (4501, 8572)
		(2, 1)	801	3123	0.711	0.046	3.00 (2.97, 3.04)	0.715	0.048	3.00 (2.91, 3.09)	3123 (2896, 3382)	0.802	0.048	3.96 (3.61, 4.51)	6550 (5099, 9098)

*Note:* Power is evaluated using 2000 simulated trials. Type I error rate is evaluated using another 2000 simulated trials under $\beta_{0}=0$.

^a^ The sample size *n* and the number of events *L* under the fixed design are calculated with parameters $\left\{\nu ,\theta ,(\tau_{a},\tau_{f})\right\}$ to achieve target power 0.8 under a treatment effect $\beta_{0}=\log(0.8)$ and a two‐sided significance level 0.05.

To conclude, the results from different event‐generating processes and heterogeneity justified the use of ${\hat{v}}_{\text{blind}}^{2}$ in the form of (13) to monitor the predicted power of clinical trials with recurrent events. With sample sizes derived from correctly specified parameters at the design stage, the proposed method achieved the desirable level of power, albeit with a slightly longer trial duration.

### 4.4 Performance of the Proposed Monitoring Procedure Under Parameter Misspecification at the Design Stage

Table 2 shows the power and analysis time for the fixed design, re‐estimation method, and our proposed method across the three misspecification scenarios introduced in Section 4.2. In scenario‐1, we calculated the sample size n=281, and the target number of events for the fixed design was 631. Under the fixed design, as $\theta_{\text{true}}$ increased, we observed a decrease in power from 0.801 to 0.562. The re‐estimation procedure of the frailty variance mitigated the impact of ignoring heterogeneity on power. In the worst case, the re‐estimation procedure achieved an empirical power of 0.686, with the re‐estimated target number of events being 1060 (Q1, Q3: 1004, 1148). In contrast, by applying the proposed blinded monitoring of the robust variance, the empirical power was maintained near 0.8 at the cost of prolonging the total follow‐up period, with the longest median analysis time being 8.60 years (Q1, Q3: 7.14, 10.63) and the number of events at the final analysis being 2028 (Q1, Q3: 1679, 2540). In scenario‐2, when $\theta_{\text{assumed}}<\theta_{\text{true}}=0.5$ (underestimated case), the power under the fixed design was 0.711 and 0.753. The re‐estimation procedure achieved power of 0.748 and 0.774. On the other hand, the proposed method recovered the power around 0.8. With $\theta_{\text{assumed}}=0.6$ or 0.7 (overestimated case), the target L was still achieved at 3 years, but this resulted in increased empirical power of 0.840 and 0.865 under the fixed design. Notably, both the re‐estimation and the proposed blinded monitoring procedure mitigated the overpowering with the median analysis time shorter than the initially planned 3 years. However, the re‐estimation procedure still resulted in a slightly higher empirical power, up to 0.834. On the other hand, the median time of the proposed method was about 2.58 years or less, along with a smaller number of events when conducting the final analysis. The results of scenario‐3 showed that misspecification of the baseline rate function would affect trial durations across all three methods. Nevertheless, by monitoring the robust variance and thus the predicted power, we controlled the power to be closer to 0.8 than its two comparators.

**TABLE 2:** The empirical power and analysis timing of the proposed monitoring procedure compared with a fixed event‐driven design and a re‐estimation procedure when sample size planning assumptions are misspecified at the design stage.

$\theta_{\text{true}}$	$\theta_{\text{assumed}}$	*n* ^a^	*L* ^a^	Ingel and Jahn‐Eimermacher [18]					Proposed		
				Fixed		Re‐estimation			Monitoring		
					Analysis time: Median (Q1, Q3)		Analysis time: Median (Q1, Q3)			Analysis time: Median (Q1, Q3)	
				Power	Calendar time (years)	Power	Calendar time (years)	# of events	Power	Calendar time (years)	# of events
Scenario‐1											
0^b^	0	281	631	0.801	3.00 (2.93, 3.06)	0.813	3.05 (2.96, 3.16)	634 (632, 661)	0.802	3.03 (2.87, 3.20)	641 (604, 678)
0.1				0.721	2.99 (2.93, 3.07)	0.779	3.59 (3.34, 3.81)	783 (739, 831)	0.799	3.79 (3.53, 4.08)	829 (768, 903)
0.2				0.641	2.99 (2.91, 3.08)	0.735	4.18 (3.90, 4.43)	932 (866, 991)	0.814	5.18 (4.66, 5.79)	1181 (1062, 1339)
0.3				0.562	3.00 (2.91, 3.09)	0.686	4.71 (4.44, 5.06)	1060 (1004, 1148)	0.796	8.60 (7.14, 10.63)	2028 (1679, 2540)
Scenario‐2											
0.5	0.3	473	1063	0.711	3.00 (2.93, 3.08)	0.748	3.65 (3.45, 3.88)	1346 (1263, 1428)	0.807	5.16 (4.48, 6.04)	1975 (1687, 2354)
	0.4	536	1206	0.753	3.00 (2.93, 3.07)	0.774	3.33 (3.14, 3.54)	1367 (1283, 1463)	0.795	3.83 (3.43, 4.29)	1603 (1420, 1826)
	0.5^b^	600	1350	0.808	3.00 (2.93, 3.07)	0.802	3.04 (2.85, 3.21)	1367 (1275, 1461)	0.814	3.05 (2.81, 3.35)	1374 (1247, 1538)
	0.6	664	1494	0.840	3.00 (2.94, 3.06)	0.812	2.80 (2.64, 2.96)	1373 (1283, 1467)	0.799	2.58 (2.40, 2.79)	1243 (1134, 1368)
	0.7	728	1638	0.865	3.00 (2.94, 3.07)	0.834	2.59 (2.43, 2.75)	1363 (1268, 1469)	0.794	2.25 (2.12, 2.41)	1145 (1067, 1243)

$\nu_{\text{true}}$	$\nu_{\text{assumed}}$	*n* ^a^	*L* ^a^	Ingel and Jahn‐Eimermacher [18]					Proposed		
				Fixed		Re‐estimation			Monitoring		
				Power	Analysis time: Median (Q1, Q3)	Power	Analysis time: Median (Q1, Q3)		Power	Analysis time: Median (Q1, Q3)	
					Calendar time (years)		Calendar time (years)	# of events		Calendar time (years)	# of events
Scenario‐3											
0.5	1	600	1350	0.791	6.76 (6.43, 7.09)	0.785	6.91 (6.01, 7.93)	1369 (1273, 1465)	0.793	6.77 (5.63, 8.36)	1354 (1225, 1511)
1^b^				0.808	3.00 (2.93, 3.07)	0.802	3.04 (2.85, 3.21)	1367 (1275, 1461)	0.814	3.05 (2.81, 3.35)	1374 (1247, 1538)
2				0.772	2.05 (2.03, 2.08)	0.772	2.05 (2.00, 2.11)	1344 (1258, 1436)	0.797	2.18 (2.08, 2.28)	1560 (1391, 1767)

*Note:* Power is evaluated using 2000 simulated trials under $\beta_{0}=\log(0.8)$.

^a^ The sample size *n* and the number of events for the fixed design are calculated using $\left\{\nu =1,\theta_{\text{assumed}},(\tau_{a},\tau_{f})=(1,2)\right\}$ under scenario‐1 and scenario‐2 or $\left\{\nu_{\text{assumed}},\theta =0.5,(\tau_{a},\tau_{f})=(1,2)\right\}$ under scenario‐3 with $\beta_{0}=\log(0.8)$, and allocation ratio 1:1 for a target power 0.8 and a two‐sided significance level 0.05.

^b^ The situation in which all sample size planning assumptions are correctly specified.

The re‐estimation procedure had improved performance over the fixed design. However, its performance was not satisfactory, with poor achievement of the target power. The re‐calculation by Ingel and Jahn‐Eimermacher [18] was based on an updated estimate of the frailty variance, while relying on the same setting for $\mu_{0}(\bar{\tau })$. Then, $L_{2}$ could be poorly estimated. On the other hand, our approach can use an updated estimate of the baseline mean function $\mu_{0}(t)$ up to τ, the maximum of all observed follow‐up durations. This explains why the re‐estimation procedure could only partially recover the target power under misspecified planning assumptions.

In summary, the proposed method can protect the statistical power of clinical trials with recurrent events from not only underestimation but also overestimation of sample sizes. Based on the target value of the robust variance derived at the design stage, the proposed monitoring procedure minimizes the impact of misspecified planning assumptions by adjusting the follow‐up durations.

### 4.5 Impact of Non‐Administrative Censoring

Recall that only administrative censoring was considered in the simulation study in the previous sections. To evaluate the performance of the proposed procedure under a more realistic censoring mechanism, we conducted an additional simulation study incorporating non‐administrative censoring. In the previous simulation study, subjects were followed only until the administrative end of the trial. However, in practice, non‐administrative censoring occurs when a subject drops out unexpectedly due to consent withdrawal, occurrence of adverse events, and so on.

For each subject i, we generated an additional censoring time $C_{i}$ from an exponential distribution with rate parameter $\lambda_{c}$, independently of the recurrent event process and treatment assignment. The observed follow‐up time $C_{i}^{obs}$ was then defined as the minimum of the administrative follow‐up time and $C_{i}$. Since, the administrative end in the previous simulation was 15 years, $C_{i}^{obs}=\min(15-r_{i},C_{i})$, where $r_{i}$ was the entry time of the ith subject following the uniform distribution on $\left[0,\tau_{a}\right]$. Event times for subject i were generated up to the corresponding observed follow‐up time $C_{i}^{obs}$. The rate parameter $\lambda_{c}$ was calibrated so that the marginal probability of non‐administrative censoring at the planned end of calendar year 3 was 3%, while accounting for staggered entry. Under this calibration, the proportion of subjects lost to follow‐up before the administrative end ranged from 16.2% to 19.2% across three different accrual and follow‐up schemes. Using this censoring mechanism, we repeated the two simulation comparisons under the same correctly specified and misspecified settings as considered in Section 4.2.

The results showed that the proposed procedure worked well even in the presence of non‐administrative censoring. Compared with the administrative censoring‐only setting, the main effect of incorporating non‐administrative censoring was a modest delay in the calendar year to the final analysis and a slight increase in the number of events required at final analysis in many scenarios. In the correctly specified cases as presented in Table 3, with ν=2 and θ>0, the same pattern remained but became more pronounced. For example, when $\nu =2,\theta =1,(\tau_{a},\tau_{f})=(1,2)$, the median calendar year for the proposed method increased from 3.28 to 3.41 years and the number of events at the final analysis also increased from 5282 (Q1, Q3: 3890, 7776) to 5629 (Q1, Q3: 4099, 8470). A similar pattern was also observed in the misspecified settings as shown in Table 4.

**TABLE 3:** The impact of non‐administrative censoring on the operating characteristics of the proposed monitoring procedure, compared with a fixed event‐driven design and a re‐estimation procedure when sample size planning assumptions are correctly specified at the design stage.

ν	θ	$\left(\tau_{a},\tau_{f}\right)$	*n* ^a^	*L* ^a^	Ingel and Jahn‐Eimermacher [18]							Proposed			
					Fixed			Re‐estimation				Monitoring			
					Power	Type I error	Analysis time: Median (Q1, Q3)	Power	Type I error	Analysis time: Median (Q1, Q3)		Power	Type I error	Analysis time: Median (Q1, Q3)	
							Calendar time (years)			Calendar time (years)	# of events			Calendar time (years)	# of events
0.5	0	(1, 2)	445	631	0.794	0.054	3.04 (2.91, 3.19)	0.802	0.054	3.12 (2.96, 3.32)	633 (632, 636)	0.799	0.058	3.10 (2.83, 3.40)	637 (608, 668)
		(1.5, 1.5)	470	631	0.793	0.054	3.04 (2.92, 3.16)	0.803	0.054	3.10 (2.96, 3.28)	633 (632, 635)	0.795	0.060	3.07 (2.84, 3.33)	636 (607, 666)
		(2, 1)	502	631	0.796	0.054	3.04 (2.93, 3.15)	0.798	0.054	3.08 (2.96, 3.22)	633 (632, 634)	0.792	0.056	3.06 (2.85, 3.28)	635 (608, 663)
	0.5	(1, 2)	764	1085	0.798	0.048	3.05 (2.91, 3.20)	0.789	0.046	3.08 (2.75, 3.45)	1093 (1029, 1157)	0.794	0.049	3.07 (2.67, 3.53)	1083 (1002, 1178)
		(1.5, 1.5)	790	1061	0.797	0.056	3.05 (2.93, 3.18)	0.803	0.056	3.09 (2.83, 3.41)	1072 (1014, 1132)	0.800	0.052	3.12 (2.77, 3.52)	1075 (999, 1160)
		(2, 1)	822	1034	0.795	0.050	3.04 (2.93, 3.14)	0.797	0.054	3.09 (2.85, 3.35)	1046 (993, 1103)	0.803	0.051	3.15 (2.86, 3.46)	1061 (991, 1134)
	1	(1, 2)	1084	1539	0.793	0.046	3.05 (2.92, 3.20)	0.798	0.048	3.08 (2.78, 3.46)	1550 (1459, 1647)	0.797	0.050	3.12 (2.63, 3.78)	1553 (1405, 1735)
		(1.5, 1.5)	1109	1490	0.790	0.053	3.06 (2.94, 3.18)	0.791	0.048	3.08 (2.82, 3.39)	1497 (1418, 1587)	0.796	0.050	3.14 (2.70, 3.67)	1513 (1379, 1667)
		(2, 1)	1141	1436	0.786	0.050	3.04 (2.93, 3.15)	0.792	0.057	3.07 (2.84, 3.34)	1448 (1371, 1525)	0.799	0.054	3.17 (2.80, 3.58)	1475 (1356, 1616)
1	0	(1, 2)	281	631	0.802	0.048	3.03 (2.96, 3.11)	0.810	0.048	3.08 (3.00, 3.20)	634 (632, 661)	0.803	0.046	3.07 (2.90, 3.25)	642 (603, 682)
		(1.5, 1.5)	312	631	0.785	0.056	3.04 (2.97, 3.10)	0.796	0.056	3.08 (3.00, 3.18)	633 (632, 659)	0.790	0.059	3.07 (2.93, 3.22)	640 (605, 679)
		(2, 1)	351	631	0.793	0.048	3.04 (2.97, 3.09)	0.799	0.048	3.08 (3.00, 3.16)	633 (632, 655)	0.794	0.048	3.06 (2.93, 3.19)	639 (603, 674)
	0.5	(1, 2)	600	1350	0.797	0.043	3.04 (2.97, 3.11)	0.797	0.043	3.07 (2.88, 3.27)	1367 (1276, 1468)	0.801	0.044	3.13 (2.87, 3.48)	1400 (1262, 1573)
		(1.5, 1.5)	632	1278	0.787	0.051	3.03 (2.97, 3.10)	0.793	0.052	3.07 (2.90, 3.24)	1294 (1213, 1378)	0.800	0.052	3.16 (2.92, 3.42)	1337 (1218, 1483)
		(2, 1)	670	1206	0.777	0.046	3.03 (2.97, 3.09)	0.780	0.054	3.05 (2.91, 3.20)	1219 (1144, 1295)	0.797	0.054	3.22 (3.02, 3.44)	1311 (1197, 1448)
	1	(1, 2)	920	2069	0.791	0.044	3.04 (2.97, 3.11)	0.798	0.046	3.07 (2.88, 3.28)	2091 (1950, 2243)	0.798	0.052	3.16 (2.79, 3.65)	2164 (1867, 2548)
		(1.5, 1.5)	951	1925	0.782	0.054	3.04 (2.97, 3.10)	0.786	0.051	3.06 (2.90, 3.23)	1940 (1821, 2079)	0.792	0.052	3.24 (2.92, 3.65)	2093 (1825, 2420)
		(2, 1)	990	1781	0.778	0.059	3.03 (2.98, 3.09)	0.776	0.056	3.05 (2.90, 3.19)	1793 (1678, 1908)	0.801	0.056	3.34 (3.06, 3.66)	2032 (1803, 2331)
2	0	(1, 2)	111	631	0.783	0.054	3.02 (2.98, 3.07)	0.796	0.052	3.08 (3.02, 3.17)	646 (632, 707)	0.798	0.056	3.08 (2.94, 3.23)	657 (597, 729)
		(1.5, 1.5)	134	631	0.790	0.062	3.02 (2.98, 3.07)	0.804	0.060	3.06 (3.01, 3.14)	643 (632, 682)	0.798	0.060	3.06 (2.94, 3.18)	653 (592, 717)
		(2, 1)	162	631	0.791	0.056	3.03 (2.98, 3.07)	0.801	0.054	3.07 (3.01, 3.13)	636 (632, 680)	0.794	0.060	3.06 (2.95, 3.18)	651 (596, 710)
	0.5	(1, 2)	431	2452	0.786	0.056	3.03 (2.99, 3.06)	0.782	0.054	3.03 (2.90, 3.16)	2466 (2239, 2701)	0.796	0.060	3.25 (2.95, 3.68)	2880 (2308, 3870)
		(1.5, 1.5)	454	2141	0.773	0.044	3.03 (2.99, 3.06)	0.764	0.044	3.04 (2.93, 3.14)	2163 (1986, 2342)	0.808	0.044	3.45 (3.17, 3.84)	2972 (2417, 3887)
		(2, 1)	482	1877	0.722	0.054	3.02 (2.99, 3.06)	0.720	0.054	3.03 (2.93, 3.13)	1878 (1740, 2038)	0.790	0.058	3.65 (3.35, 4.01)	3079 (2452, 3937)
	1	(1, 2)	750	4273	0.782	0.052	3.03 (2.99, 3.06)	0.778	0.050	3.03 (2.91, 3.15)	4270 (3903, 4654)	0.800	0.052	3.41 (2.98, 4.09)	5629 (4099, 8470)
		(1.5, 1.5)	773	3650	0.745	0.051	3.03 (2.99, 3.06)	0.744	0.048	3.03 (2.92, 3.13)	3654 (3358, 3946)	0.789	0.052	3.74 (3.33, 4.42)	6175 (4650, 9222)
		(2, 1)	801	3123	0.712	0.050	3.03 (2.99, 3.06)	0.707	0.050	3.02 (2.94, 3.12)	3118 (2888, 3363)	0.794	0.054	4.10 (3.68, 4.78)	6902 (5199, 10046)

*Note:* Power is evaluated using 2000 simulated trials. Type I error rate is evaluated using another 2000 simulated trials under $\beta_{0}=0$.

^a^ The sample size *n* and the number of events *L* under the fixed design are calculated with parameters $\left\{\nu ,\theta ,(\tau_{a},\tau_{f})\right\}$ to achieve target power 0.8 under a treatment effect $\beta_{0}=\log(0.8)$ and a two‐sided significance level 0.05.

**TABLE 4:** The impact of non‐administrative censoring on the empirical power and analysis timing of the proposed monitoring procedure compared with a fixed event‐driven design and a re‐estimation procedure when sample size planning assumptions are misspecified at the design stage.

$\theta_{\text{true}}$	$\theta_{\text{assumed}}$	*n* ^a^	*L* ^a^	Ingel and Jahn‐Eimermacher [18]					Proposed		
				Fixed		Re‐estimation			Monitoring		
				Power	Analysis time: Median (Q1, Q3)	Power	Analysis time: Median (Q1, Q3)		Power	Analysis time: Median (Q1, Q3)	
					Calendar time (years)		Calendar time (years)	# of events		Calendar time (years)	# of events
Scenario‐1											
0^b^	0	281	631	0.802	3.03 (2.96, 3.11)	0.810	3.08 (3.00, 3.20)	634 (632, 661)	0.803	3.07 (2.90, 3.25)	642 (603, 682)
0.1				0.735	3.03 (2.96, 3.11)	0.805	3.68 (3.41, 3.90)	787 (739, 833)	0.830	3.88 (3.58, 4.21)	836 (768, 912)
0.2				0.627	3.04 (2.95, 3.12)	0.723	4.27 (3.98, 4.54)	931 (861, 990)	0.793	5.42 (4.84, 6.13)	1208 (1067, 1373)
0.3				0.554	3.03 (2.94, 3.13)	0.673	4.81 (4.52, 5.16)	1054 (1000, 1139)	0.774	9.43 (7.50, 12.37)	2144 (1713, 2776)
Scenario‐2											
0.5	0.3	473	1063	0.697	3.04 (2.96, 3.12)	0.744	3.71 (3.49, 3.94)	1341 (1259, 1422)	0.802	5.43 (4.60, 6.48)	2032 (1705, 2442)
	0.4	536	1206	0.743	3.04 (2.96, 3.12)	0.761	3.39 (3.19, 3.61)	1370 (1286, 1463)	0.797	3.93 (3.51, 4.44)	1617 (1423, 1849)
	0.5^b^	600	1350	0.797	3.04 (2.97, 3.11)	0.797	3.07 (2.88, 3.27)	1367 (1276, 1468)	0.801	3.13 (2.87, 3.48)	1400 (1262, 1573)
	0.6	664	1494	0.838	3.04 (2.97, 3.10)	0.817	2.81 (2.64, 3.00)	1363 (1273, 1464)	0.794	2.62 (2.43, 2.85)	1250 (1146, 1379)
	0.7	728	1638	0.877	3.04 (2.97, 3.10)	0.843	2.61 (2.45, 2.78)	1366 (1268, 1475)	0.814	2.28 (2.14, 2.45)	1157 (1069, 1258)

$\nu_{\text{true}}$	$\nu_{\text{assumed}}$	*n* ^a^	*L* ^a^	Ingel and Jahn‐Eimermacher [18]					Proposed		
				Fixed		Re‐estimation			Monitoring		
				Power	Analysis time: Median (Q1, Q3)	Power	Analysis time: Median (Q1, Q3)		Power	Analysis time: Median (Q1, Q3)	
					Calendar time (years)		Calendar time (years)	# of events		Calendar time (years)	# of events
Scenario‐3											
0.5	1	600	1350	0.800	7.08 (6.72, 7.48)	0.799	7.25 (6.26, 8.35)	1364 (1272, 1460)	0.800	7.27 (5.91, 9.15)	1361 (1231, 1521)
1^b^				0.797	3.04 (2.97, 3.11)	0.797	3.07 (2.88, 3.27)	1367 (1276, 1468)	0.801	3.13 (2.87, 3.48)	1400 (1262, 1573)
2				0.776	2.07 (2.04, 2.09)	0.774	2.07 (2.01, 2.13)	1353 (1262, 1440)	0.812	2.20 (2.10, 2.32)	1579 (1407, 1804)

*Note:* Power is evaluated using 2000 simulated trials under $\beta_{0}=\log(0.8)$.

^a^ The sample size *n* and the number of events for the fixed design are calculated using $\left\{\nu =1,\theta_{\text{assumed}},(\tau_{a},\tau_{f})=(1,2)\right\}$ under scenario‐1 and scenario‐2 or $\left\{\nu_{\text{assumed}},\theta =0.5,(\tau_{a},\tau_{f})=(1,2)\right\}$ under scenario‐3 with $\beta_{0}=\log(0.8)$, and allocation ratio 1:1 for a target power 0.8 and a two‐sided significance level 0.05.

^b^ The situation in which all sample size planning assumptions are correctly specified.

## 5 Example

In this section, we illustrate the proposed blinded monitoring procedure for determining the timing of final analysis using chronic granulomatous disease (CGD) data, which are available in the *R* package survival. The International Chronic Granulomatous Disease Cooperative Study Group designed a multicenter, randomized, double‐blind trial to evaluate the efficacy of interferon gamma in preventing severe infections in patients with CGD. In the original study design, subjects were randomized to receive either placebo or interferon gamma for 12 months. The primary endpoint was the time to the first severe infection, and multiple times to serious infection were also recorded [29]. The trial was terminated early following an interim analysis in July 1989. A total of 128 eligible patients were enrolled in the trial, with 65 assigned to the placebo group and 63 to the interferon gamma group [30].

We used multiple times to serious infections as the recurrent events of interest for illustration. To derive the target number of events at the design stage, we used formula (10). In reality, we face difficulty in setting relevant parameters for the mixed Poisson model and may use existing datasets to this end. Here, for illustrative purposes, we set values for parameters in the model that properly fit the placebo group of the CGD data. As described above, the planned follow‐up duration $\bar{\tau }$ was 1 year. Assuming a constant baseline intensity, the estimated ${\hat{\lambda }}_{0}$ was 1.10 events/(person · year). Applying an EM algorithm [31] to the placebo group, we estimated the frailty variance $\hat{\theta }$ to be 0.777. With a target power 1−γ=0.8, a two‐sided significance level α=0.05, and a prespecified treatment effect $\beta_{0}=\log(0.3)$, we set the target L=38 for the fixed design. Here, we calculated the target number of events under the correctly specified planning assumptions. Thus, the comparison with the re‐estimation procedure was skipped. In this illustration, we set n=128. That is, we would conduct the final analysis if we collected 38 events from the 128 patients in the fixed design.

We explain the proposed method following the steps in Section 3.3. In Step 1 (see Section 3.3), we set the two‐sided significance level, the target power, and the target treatment effect to the same values as in the fixed design described above. In Step 2, we did not follow the procedure in Section 3.3. Instead, we also set n=128 for our proposed procedure. In Step 3, we obtained the target variance $v_{\text{target}}^{2}=0.185$ for the blinded monitoring procedure. The dataset contains the randomization date for each subject. In this illustration, the first date of randomization was considered the date of study initiation. The trial lasted from August 28, 1988, to the final follow‐up date of January 17, 1990, covering 507 days. Monitoring began one month after the trial was initiated, and thereafter we evaluated five intervals to monitor the blinded estimator of the robust variance ${\hat{v}}_{\text{blind}}^{2}$ or the corresponding predicted power: Daily (continuous), weekly, monthly, every 2 months, and every 4 months in Step 4. At the first point when the predicted power exceeded 0.8 (as in Step 5), we fitted the marginal rate model to estimate β along with its robust standard error and corresponding *p* value from the robust Wald test.

An aspect of applying our proposed method is that the quantity ${\hat{v}}_{\text{blind}}^{2}$, or the resulting predicted power, is an estimate and therefore subject to uncertainty in the estimation. To visualize such uncertainty and facilitate the decision to final analysis accounting for it, we applied a bootstrap approach [32] to compute confidence intervals for ${\hat{v}}^{2}$ during monitoring. For each resampled dataset, we computed ${\hat{v}}^{2}$ at each monitoring time point τ,τ∈{τ∈ℤ|30≤τ≤507}. With 2000 bootstrap samples, we obtained the distribution of ${\hat{v}}^{2}$ at each τ and formed the 95% confidence intervals using the percentile approach.

In Table 5, we present the results of the statistical analysis based on the fixed design and the monitoring procedure. In the fixed design, we conducted the final analysis on day 306 from the start of the study. The rate ratio was estimated as 0.294 (95% CI: 0.124−0.700) with P=0.006. The proposed continuous (daily) monitoring procedure suggested conducting the final analysis a little earlier on day 281 with the predicted power of 0.802. The rate ratio was estimated as 0.294 (95% CI: 0.124−0.699) with P=0.006. When following a longer interval for the monitoring procedure, the time of final analyses might be delayed, and perhaps with a higher predicted power. Compared with continuous (daily) monitoring, the time to final analysis for a 4‐month interval (Day 390) increased by 38.8%, and the corresponding predicted power was 0.951. In general, longer monitoring intervals tend to delay the final analysis because the stopping criterion is checked less frequently. However, because monitoring is performed on a discrete calendar schedule, the effect of less frequent monitoring depends not only on the nominal interval length but also on how the review dates align with the realized trajectory of the monitored variance.

**TABLE 5:** Results of CGD data under $\beta_{0}=\log(0.3)$ for a target power of 0.8 and two‐sided significance level of 0.05: fixed design vs. proposed monitoring procedure.

	Fixed	Proposed				
		Continuous	Weekly	Monthly	2‐month	4‐month
Calendar time (days)	306	281	289	300	330	390
# of events	38	33	36	36	46	70
Predicted power^a^	NA	0.802	0.826	0.823	0.853	0.951
$\hat{\beta }$ (SE)	−1.223 (0.442)	−1.224 (0.441)	−1.192 (0.449)	−1.145 (0.444)	−1.333 (0.433)	−1.128 (0.329)
$\exp\hat{\left(\beta \right)}$ (95% CI)	0.294 (0.124, 0.700)	0.294 (0.124, 0.699)	0.303 (0.126, 0.731)	0.318 (0.133, 0.760)	0.264 (0.113, 0.617)	0.324 (0.170, 0.617)
*p*	0.006	0.006	0.008	0.010	0.002	0.001

Abbreviations: CI, confidence interval; NA, not applicable; SE, standard error.

^a^ The predicted power is monitored using the power formula (12). For the proposed monitoring procedure, on the day the predicted power exceeded the target power of 0.8 for the first time, we conducted the final analysis.

**FIGURE 1:**
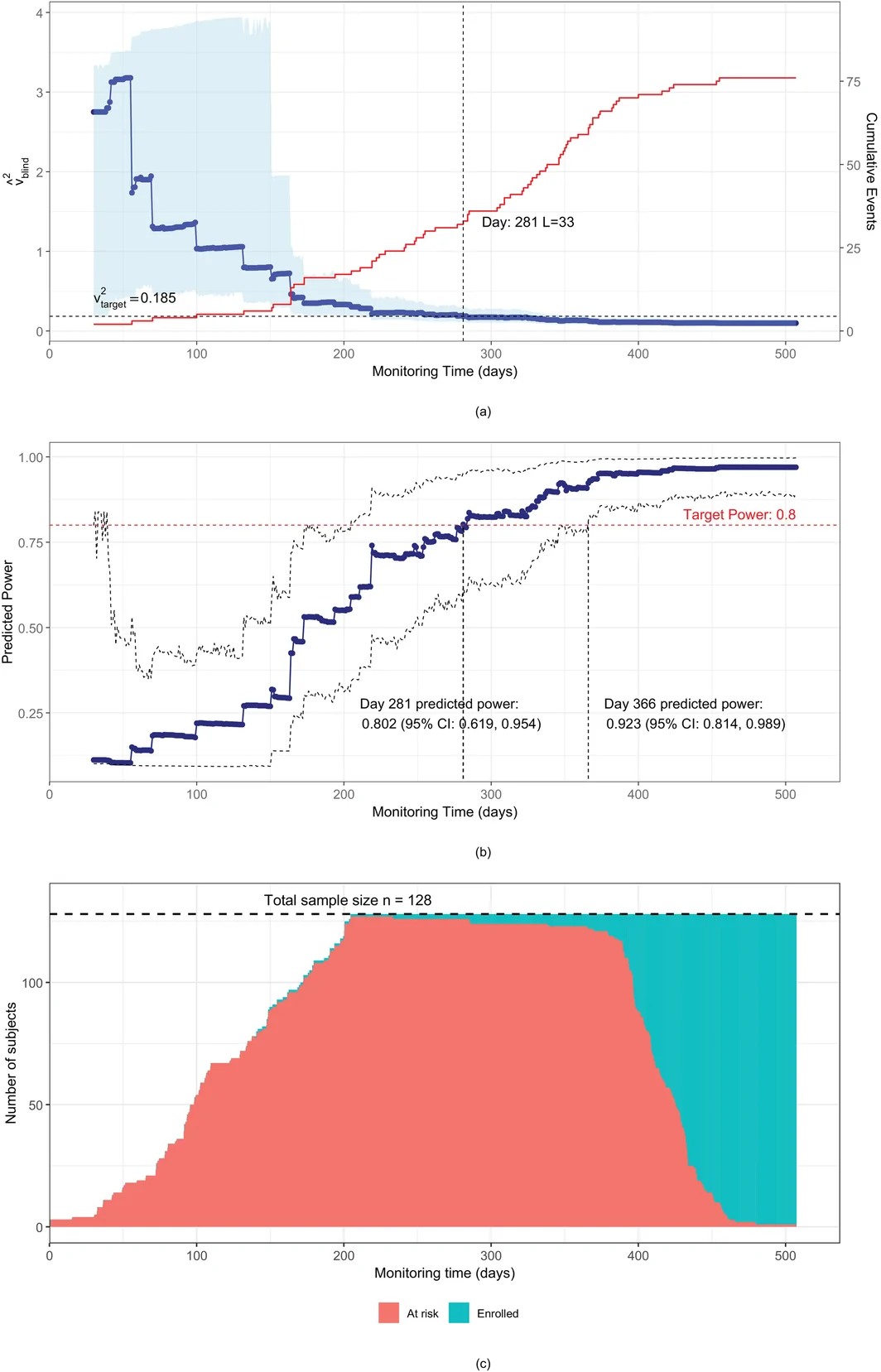
(a) shows the changes in ${\hat{v}}_{\text{blind}}^{2}$ (dark blue curve) over monitoring time, with the light blue shaded region representing the bootstrapped confidence interval for ${\hat{v}}_{\text{blind}}^{2}$. The red curve shows changes in event counts over time. (b) shows the transformed predicted power and corresponding bootstrapped upper and lower bounds using Equation (12) with $\beta_{0}=\log(0.3)$ and two‐sided significance level α=0.05 over monitoring time. (c) shows the changes in the number of subjects enrolled and the number of subjects at risk over monitoring time.

Figure 1 illustrates the changes of the blinded estimator ${\hat{v}}_{\text{blind}}^{2}$, the corresponding predicted power, and the size of the risk set as the trial progresses. In Figure 1a, we present the cumulative number of events L in both groups over the calendar time (the curve in red) and the blinded variance estimator ${\hat{v}}_{\text{blind}}^{2}$ (the dark blue curve) with pointwise bootstrap 95% confidence intervals (the light‐blue shaded areas). The vertical black dashed line marks day 281, when the predicted power first reached 0.8 under continuous monitoring; 33 events had been observed by that time. Besides, at the early stage of the trial, the estimator fluctuated substantially. The bootstrap CIs were wide, implying a limited amount of information available when follow‐up was short and only a small number of events had been observed. After six months, as more events accumulated, the blinded estimator became smoother, and the CIs narrowed, indicating increasing stability of ${\hat{v}}_{\text{blind}}^{2}$. However, this timing may not be interpreted as universal. The time at which ${\hat{v}}_{\text{blind}}^{2}$ becomes sufficiently stable depends on multiple factors, including the number of observed events early in follow‐up, the overall event accumulation rate, and the degree of heterogeneity in the recurrent event process. Alternatively, we converted ${\hat{v}}_{\text{blind}}^{2}$ to predicted power in Figure 1b. Specifically, we estimated the power 0.802 (95% CI: 0.619−0.954) on day 281 for continuous monitoring. As shown in the simulation studies and in this example, the proposed monitoring procedure based on the point estimate of ${\hat{v}}_{\text{blind}}^{2}$ works well under various scenarios. In practice, the CIs can act as a supplementary tool to assess whether the stopping criterion ${\hat{v}}_{\text{blind}}^{2}\leq v_{\text{target}}^{2}$ appears stable or may be due to random fluctuation. They may also support a more conservative decision if investigators prefer. For example, one could extend the follow‐up until day 366. At this point, with 67 events, the lower bound of the predicted power exceeded 0.8, and the point estimate reached 0.923 (95% CI: 0.814−0.989). However, such a strategy should be used with caution, as it will prolong the trial. As shown in the example, the time to final analysis increased by 30.2% compared with the strategy that uses the point estimate.

Furthermore, Figure 1c shows the number of subjects recruited and at risk over monitoring time in the CGD trial. A concern in the proposed method is that it may take too long to achieve the target variance. Continued follow‐up may provide little additional information if most subjects are censored. Therefore, monitoring the number of subjects at risk may help assess whether the target variance is likely to be reached. For example, around day 250, the target variance had not been reached. With Figure 1c, many subjects remained at risk, suggesting that further follow‐up could still provide sufficient information to attain the target variance.

These results demonstrate that the monitoring procedure allows for earlier evaluation, potentially reducing trial duration without compromising the accuracy of the treatment effect estimate. Thus, compared to the conventional fixed design, the monitoring procedure offers higher operational efficiency, greater flexibility, and timely decision‐making.

## 6 Discussion

In this paper, we proposed an event‐driven type design for clinical trials with recurrent events. Under a local alternative hypothesis, we derived a robust variance estimator free of treatment allocation. Instead of determining the timing of the statistical analysis solely by the number of events, as in standard event‐driven studies, we monitored this proposed estimator under blinded treatment allocation. We determined the timing of the statistical analysis when the monitored variance reached the target variance. To the best of our knowledge, this is the first realization of an event‐driven type design for recurrent events that explicitly accounts for within‐subject dependence through blinded monitoring of the sandwich variance under a semiparametric marginal rate model.

We would like to discuss some practical issues in implementing the proposed method. Continuous monitoring is ideal for implementing our method. However, it may not be feasible in practice. If this is the case, periodic monitoring with certain short intervals, like weekly and monthly ones, would be a practical solution. If timely monitoring at short intervals is infeasible, it is desirable to evaluate the potential delay in the analysis timing. Event prediction for recurrent events could help address the issue. In the context of non‐recurrent events, Chen et al. [33] used parametric mixture cure rate models to predict final analysis timing in event‐driven studies with cancer immunotherapy, in which delayed treatment effect and long‐term survivors may slow event accumulation. However, such a prediction is more challenging for recurrent events due to within‐subject correlation. Ren and colleagues [34] proposed Bayesian prediction frameworks based on joint frailty models to predict the timing of observing a target number of recurrent events in the presence of terminal events.

Another issue is how to handle unacceptable prolongation of the trial. There are several possibilities for it. One possibility is that subjects' enrollment is progressing much more slowly than expected. Enrollment and event prediction [35, 36] would be helpful in such a situation. Another possibility of unacceptable prolongation is severe non‐administrative censoring. As illustrated in the Example section with Figure 1c, monitoring the number at risk would be useful to determine whether one should wait for the formal attainment of the target variance or conduct the statistical analysis earlier. The predicted power would be helpful for decision‐making. If the predicted power is close to the target, the study team may decide to conclude the trial without formal attainment of the target power. If it is far from the target power, further follow‐up or other adaptations would require careful consideration, possibly with support from the event prediction. A practical solution to avoid substantial delays in statistical analysis is to set a prespecified maximum follow‐up duration. Careful consideration is required to define such a fixed time frame. However, unexpectedly slow enrollment of subjects may lead to a substantially underpowered study if the statistical analysis is conducted at the fixed time frame. Likewise, the predicted power would be useful in deciding whether the statistical analysis should be conducted as planned at the fixed time frame. Further methodological development to support such decisions is warranted.

In this paper, we focused on determining the timing of the statistical analysis and did not discuss how to set the sample size. Instead, we relied on the method of Ingel and Jahn‐Eimermacher [18]. However, the proposed monitoring strategy based on robust variance does not eliminate the need to choose an appropriate sample size at the design stage. If the planned sample size is too small, the time required for the monitored variance to reach the target can become substantially longer than expected. Indeed, as observed in our simulation study with substantial misspecification of certain nuisance quantities, such as the baseline rate and frailty variance at the design stage, it might lead to noticeable prolongation of the trials. If this is the case, the trial may be infeasible to complete. Even if feasible, it may cause unacceptable delays in sharing scientific findings with the public. Thus, it is essential to set an appropriate initial sample size that well reflects the true probabilistic structure of the recurrent events. Many authors have developed methods to determine sample sizes at the design stage that model within‐subject dependence, such as the mixed Poisson model [15, 17, 18]. Careful consideration of the sample size is required, possibly using some of these methods.

It would be an interesting research topic to tailor sample size calculation methods to suit our method better. The study team may wish to enroll more subjects with shorter follow‐up durations to complete the study shortly. We need to balance between the utility of decreasing the sample size and that of shortening the follow‐up duration. More generally, such consideration could be made with the possibility of mid‐trial sample size adaptation. We do not exclude this possibility, but note that several points require attention. First, additional recruitment requires further assumptions about the event rate and censoring patterns among newly enrolled subjects. Second, increasing the number of subjects may not always be possible, particularly in trials of rare diseases, where the number of eligible patients is limited. Third, the adaptation should be prespecified clearly and implemented in a manner that preserves trial integrity [37, 38]. The feasibility and timing of further accrual should be considered. For example, Ingel and Jahn‐Eimermacher [18] suggested that their re‐estimation be performed before the recruitment is completed. In addition, if the sample size re‐estimation is conducted in an unblinded manner, the re‐estimation rules and final analysis should be carefully prespecified to preserve the Type I error rate, and access to unblinded data should be restricted to minimize operational bias [39, 40].

It often takes a long time to complete a confirmatory clinical trial with recurrent events as the primary endpoint. Then, from a scientific and ethical point of view, it would be required to conduct formal interim analyses to establish efficacy early. Mütze et al. [41] showed that the independent increment structure does not hold for a sequence of the Wald‐test statistics based on the proportional rate model. Then, the alpha‐spending function approach [42] or the maximum information design cannot be taken. Mütze et al. [41] proposed a method to conduct the maximum information design and empirically showed that it can maintain the target power. To be more specific, first, the maximum information is determined to attain the target power, based on the prespecified number of interim analyses and the sequence of information fractions at which the interim analyses are conducted. Then, the Type I error rate (alpha) to spend at each interim analysis is determined by the alpha‐spending function. To implement this procedure properly, one needs to conduct each interim analysis when the information fraction is attained. Mütze et al. [41] emphasized the importance of monitoring the accumulated information during the study. The formula for the information by Mütze et al. [41] depends on the treatment allocation. Our blinded estimate of the robust variance is regarded as a blinded estimation of the information. Monitoring our blinded robust variance estimate would be more practical to determine the timing of the interim analyses. A fully integrated framework that combines the proposed method with group sequential studies for trials with recurrent events remains a promising topic for further research.

## Funding

This work was supported by the Japan Society for the Promotion of Science (JSPS) KAKENHI (Grant No. 25K03086).

## Conflicts of Interest

The authors declare no conflicts of interest.

## Data Availability

We used the publicly available CGD dataset from the survival package in R. The R code implementing the proposed event‐driven type design is available in the Event_Driven_Rec repository at https://github.com/JINGWENZHANGBiostat/Event_Driven_Rec.

## Appendix A Derivation of ${\hat{v}}_{\text{blind}}^{2}$

In this appendix, we present our approximations to Â and $\hat{\sum }$. Note that, when $\beta_{0}\to 0$, $\bar{Z}(\beta_{0},t)=S^{\left(1\right)}(\beta_{0},t)/S^{\left(0\right)}(\beta_{0},t)$ approaches π [2], and ${\left(Z-\pi \right)}^{2}=\pi (1-\pi )$. We assume independent censoring (Condition 1) and C⊥Z (Condition 2). The score function is given by

$$U(\beta_{0})=\overset{n}{\underset{i=1}{\sum }}\int_{0}^{\tau }\{Z_{i}-\bar{Z}(\beta_{0},u)\}(dN_{i}(u)-Y_{i}(u)\exp(\beta_{0}Z_{i})d\mu_{0}(u)).$$

Lin et al. [12] proved that $n^{-1/2}U(\beta_{0})$ converges in distribution to a normal distribution with mean 0 and variance ∑ as defined in (6). Under the local alternative $H_{1}:\beta_{0}=\frac{\delta }{\sqrt{n}}$, $\bar{z}(\beta_{0},u)=\pi$ and

$$\begin{array}{ll} \sum & =E\left[{\left(\int_{0}^{\tau }\{Z-\pi \}\{dN(u)-Y(u)d\mu_{0}(u)\}\right)}^{2}\right]\\ & =E\left[{\left\{Z-\pi \right\}}^{2}{\left\{N(\tau )-\mu_{0}(\tau \wedge C)\right\}}^{2}\right]. \end{array}$$

When π=1/2, it is represented as

$$\begin{array}{l} \frac{1}{4}E\left[{\left\{N(\tau )-\mu_{0}(\tau \wedge C)\right\}}^{2}\right], \end{array}$$

By replacing the expectation with the sample average and the theoretical quantities with their empirical counterparts, coupled with the fact that $A=\lim_{n\to \infty }\pi (1-\pi )\frac{L}{n}$, (13) is obtained.
